# Next‐generation sequencing identifies a novel frameshift variant in *FRMD7* in a Chinese family with idiopathic infantile nystagmus

**DOI:** 10.1002/jcla.23012

**Published:** 2019-09-08

**Authors:** Fengqi Wang, Hongzai Guan, Wenmiao Liu, Guiqiu Zhao, Shiguo Liu

**Affiliations:** ^1^ Medical Genetic Department The Affiliated Hospital of Qingdao University Qingdao China; ^2^ Prenatal Diagnosis Center The Affiliated Hospital of Qingdao University Qingdao China; ^3^ Department of Clinical Hematology Medical College of Qingdao University Qingdao China; ^4^ Department of Ophthalmology The Affiliated Hospital of Qingdao University Qingdao China

**Keywords:** *FRMD7*, idiopathic infantile nystagmus, Next‐generation sequencing, variant

## Abstract

**Background:**

Idiopathic infantile nystagmus (IIN) is a high genetically heterogeneous ophthalmic disease and is often associated with pathogenic mutations in *FRMD7* and *GPR143,* respectively. Idiopathic infantile nystagmus manifests as involuntary periodic rhythmic oscillation of the eyes in the very early life, which decreases visual acuity and affects the quality of life.

**Objective and Methods:**

The aim of our study was to reveal a possible pathogenic variant through the investigation of a Chinese Han family with IIN with an implementation of a next‐generation sequencing method. Isolated DNA analysis was followed by Sanger sequencing validation. We also performed the detailed ophthalmological examination of family members.

**Results:**

We identified a novel frameshift variant in *FRMD7* (NM_194277.2: c.1419_1422dup, p.Tyr475fs), which leads to a frameshift mutation since tyrosine (Tyr) at 475 codon of FRMD7 protein (p.Tyr475fs) and co‐segregates with IIN phenotype in this family.

**Conclusions:**

We found a novel frameshift *FRMD7* variant in a Chinese Han family, which may be causative variant for IIN and can further enrich the mutation spectrum and uncover the etiology of IIN.

## 1 INTRODUCTION

Idiopathic infantile nystagmus (IIN) is a common heterogeneous oculomotor disease, (frequency of 1/1500 live births) characterized by involuntary periodic rhythmic oscillation of the eyes in the very early life, and can result in decreased visual acuity due to the images excessively moved on the retina. The oscillatory patterns of the eye can be horizontal, vertical, and mixed, and the horizontal is the most common.^1^, ^2^, ^3^, ^4^ IIN was incidental accompanied with compensatory head posture as well as squint, ametropia, anterior segment lesions, ocular fundus diseases, myopia, hypermetropia, astigmatism, but rare with ablepsia or color deficiency. IIN usually onsets in the early 6 months after birth and accompanies all the lifetime. As the treatment for its alleviation mainly lies in surgery, the prevention for IIN is of vital importance. Idiopathic infantile nystagmus is generally inherited by autosomal dominant, autosomal recessive, or X‐linked manner. X‐linked inheritance is also associated with pathologic mutations including *FRMD7* (Xq26.2) and *GPR143* (Xp22.3), and *FRMD7* mutations are more common than *GPR143* in IIN.^5^ Apart for IIN, *FRMD7* mutations are involved in congenital motor nystagmus and *GPR143* in ocular albinism, respectively.^3^

Tarpey et al (2006) first reported 22 *FRMD7* mutations including nonsense, splice site, and missense mutations in 26 families with X‐linked idiopathic congenital nystagmus^3^ and performed functional experiments showing that these mutations destroyed the function of FRMD7. FRMD7 comes from cytoskeletal protein 4.1 superfamily (RefSeq DNA: NM_194277)^6^ and shares close amino acid sequence to FARP1 (FERM, ARH/RhoGEF, and pleckstrin domain protein 1; chondrocyte‐derived ezrin‐like protein) which plays an important role in semaphorin signaling, dendrite development, and the formation of synapses. In addition, FRMD7 participates in brainstem development in the very early life with the influence of F‐actin via activating RAC1 signaling of RhoGTPases.^3^, ^7^, ^8^ Ocular movement control center consists of three parts: visual area of cerebral cortex, vestibular cerebellum, and brain stem. *FRMD7* mutations affect neurite development in areas of ocular movement control centers (visual area of cerebral cortex, vestibular cerebellum, and brain stem),^3^ while *GPR143* mutations affect ocular oscillation due to un‐functional GPR143 protein and involve in ocular albinism, a severe visual disorder. However, clear correlation between the severity of disease and mutation types remains to be uncertain.^9^

With the development of sequencing technology, next‐generation sequencing (NGS) based on the Sanger sequence validation is getting more and more important and becomes an efficient method to detect the molecular base of Mendelian disorders. Therefore, we aimed to determine causative variant in a Chinese IIN family and clarify its possible pathogenesis, which further has a fundamental impact on genetic counseling in this family.

## 2 PATIENTS AND METHODS

### 2.1 Patients

**Figure 1:**
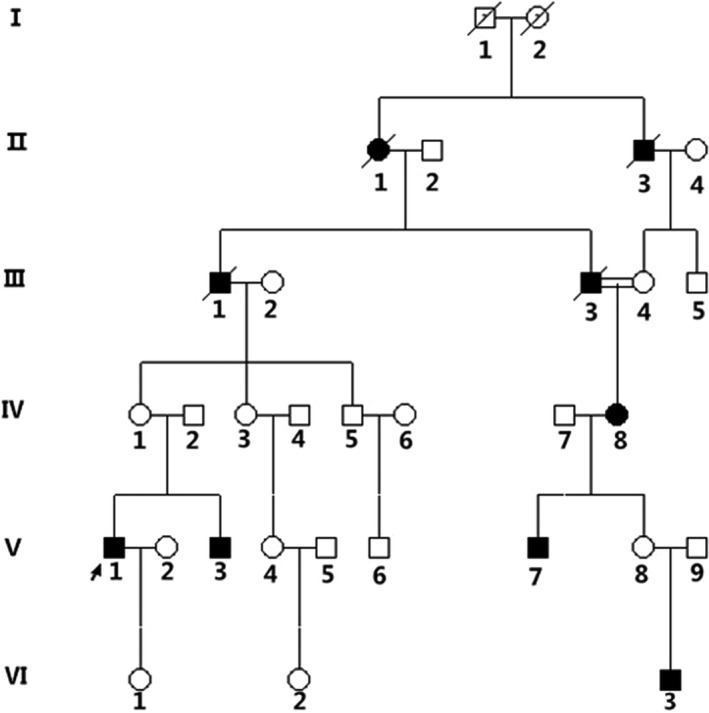
Pedigree chart of the family with Idiopathic infantile nystagmus. Circles and squares represent females and males, respectively. Black symbols indicate patients. The arrow represents the proband

This study was approved by the ethics committee of the Affiliated Hospital of Qingdao University. Subjects in this study are from a Chinese Han family (Figure 1). A 37‐year‐old male proband (Ⅴ1, Figure 1) of this family was diagnosed with IIN in ophthalmology department in the Affiliated Hospital of Qingdao University (Shandong, China) according to clinical symptoms. He suffered nystagmus few months after birth and experienced a series of detailed ophthalmic examination. This genealogy had an internuptial spouses (the sufferer Ⅲ3 and the carrier Ⅲ4, Figure 1), and they were cousins. Consanguineous marriages lead to the more frequent manifestation of diseases, especially with autosomal recessive inheritance. Blood samples were available after we obtained written informed consent of the proband and his daughter (Ⅵ1, Figure 1).

### 2.2 Methods

#### 2.2.1 Next‐generation sequencing (NGS)

Qiagen DNA extraction kit (Qiagen) was used to extract DNA samples from peripheral lymphocytes in 200 μL peripheral venous blood through the column method according to the manufacturer's instructions. Multiskan GO (Thermo Fisher) was used to determine the DNA purity and concentration. We developed the IIN capture panel based on Illumina Truseq Custom Amplicon v1.5 kit.^10^, ^11^ The IIN capture panel contains 239 genes (*NYS2, NYS3, NYS4, NYS5, NYS7, ABCA4, ABCB6, PROM1, TYR, PAX6, GPR143, SIX6, DLAT*, etc with completed coding regions) with a proven relationship to pathogenesis (genes list available as Appendix S1). Captured sample library was sequenced by Illumina Hiseq 2000 platform using V2 reagent 1.8 software. The raw image files were processed with an Illumina pipeline for base calling and stored in FastQ format (raw data). ANNOVAR was used for annotation of the variant position, variant type, and conservative prediction. Variants were filtered out according to the following criteria: (a) quality scores >30, site read depth >10×, variant depth >5×, mapping quality >50; 2) minor allele frequency (MAF) <0.01 in the 1000 Genomes databases and ExAC. To assess the pathogenicity of the variants, PolyPhen‐2, PROVEAN, CADD, and MutationTaster as alignment reference databases were analyzed by ANNOVAR and DNAMAN software version 5.0 applied with the use of GenBank reference gene sequences.

#### 2.2.2 Sanger sequencing validation

*FRMD7* variant of the proband and his daughter detected by NGS was validated further using Sanger sequencing. Two fragments covering the variant of p.Tyr475fs were amplified using *FRMD7* primer pairs for exon 12 (forward: 5′‐CACTGAGCCCAATCCTAA‐3′ and reverse 5′‐CCAACCTGCTGACCTGTA‐3′). The 20 μL PCR system contains 0.4 nmol/L of both primer pairs, 200 nmol/L dNTPs, 80 ng of template DNA, and 1.0 U AmpliTaq Gold DNA polymerase in 1 × reaction buffer (8 nmol/L Tris HCl, pH 8.3, 40 mmol/L KCl, 2.0 mmol/L MgCl2).Steps of PCR amplifications were performed as follows: initially, denaturing at 94°C for 5 minute; secondly, 35 cycles of 94°C for 30 seconds, 54°C for 30 seconds, and 72°C for 30 seconds; and finally, 10 minutes for extension at 72°C. Mutation analysis was preformed based on an automated sequencer, ABI 3730XL (Applied Biosystems) using the BigDye Terminator Cycle Sequencing kit (Applied Biosystems).

## 3 RESULTS

### 3.1 Molecular and bioinformatic analysis of *FRMD7* variant

**Figure 2:**
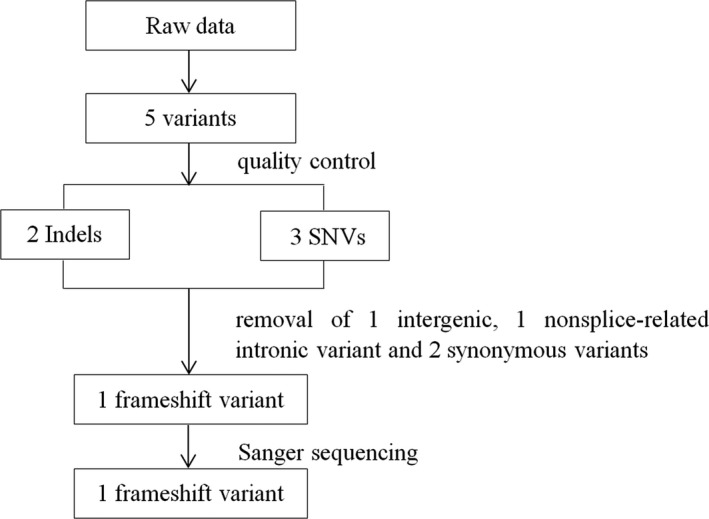
Flow chart of data analysis

**Figure 3:**
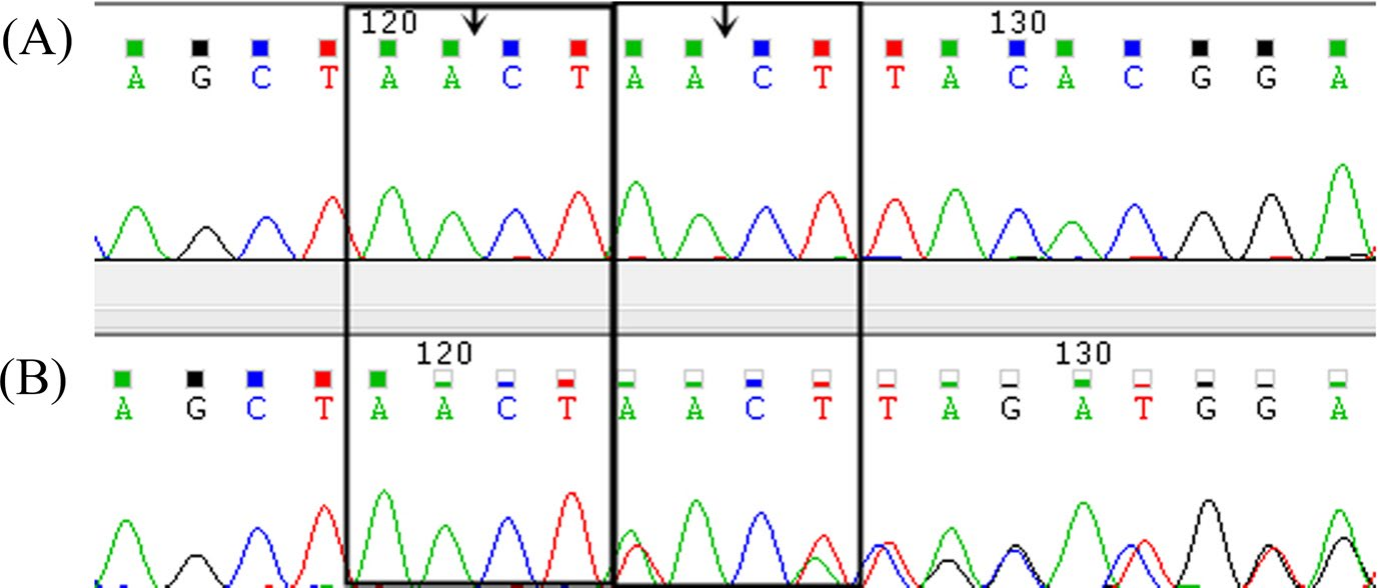
Sanger sequencing chromatograms. The male patient (A) had the novel variant, (NM_194277.2: c.1419_1422dup, p.Tyr475fs). The two rectangles indicate the location leading to frameshift variant of *FRMD7*. His daughter (B) (a carrier) is shown on the below

**Figure 4:**
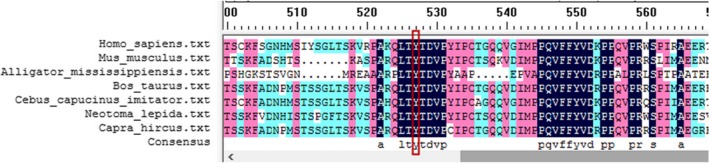
Multiple sequence alignments. Multiple sequence alignments of the FRMD7 protein including *Mus musculus, Alligator mississippiensis, Bos taurus, Cebus capucinus imitator, Neotoma lepida, and Capra hircus*. The p.Tyr475fs variant is located within a highly conserved region which shown in the rectangle

**Figure 5:**
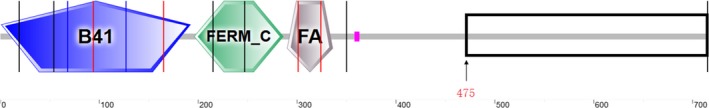
Prediction map of protein damage. The frameshift mutation resulted in translation errors from p.475Tyr and terminated at p.478, resulting in a truncated protein of 477‐amino acid

After quality control of raw data as described before, five relative credible mutations (including 2 Indels and 3 SNVs) were selected; then, intergenic and nonsplice‐related intronic variants, synonymous variants, and 3′/5′ UTR variants were excluded, and the remaining 1 frameshift mutation was verified by Sanger sequencing (Figure 2). The frameshift variant (NM_194277.2: c.1419_1422dup, p.Tyr475fs) in exon 12 of *FRMD7* (A, Figure 3) which contributed to amino acids changed (p.Tyr475fs) (Table 1). His daughter was heterozygous for this variant (B, Figure 3) but his wife has not got analyzed variant with Sanger sequencing. The depth of coverage for exon12 of *FRMD7* was 75 × and ensured high reliability of sequencing. Sanger sequencing confirmed that the proband carried this variant in exon 12 of *FRMD7*. This variant was not detected in 300 normal control individuals with the same ethnicity and is absent from gene mutation databases such as 1000 Genomes, HGMD, dbSNP, or UniProt. The FRMD7 protein sequences of *Homo sapiens, Mus musculus, Alligator mississippiensis, Bos taurus, Cebus capucinus imitator, Neotoma lepida, and Capra hircus* were obtained from NCBI, and multiple sequence alignments were performed by DANMAN software. The p.Tyr475fs variant is located in a highly conserved region of FRMD7 protein (Figure 4). Protein damage of the frameshift variant (NM_194277.2: c.1419_1422dup, p.Tyr475fs) was predicted leading to truncated protein due to changes in amino acid coding regions (Figure 5).

**Table 1:** Data of mutation site

Chromosome	Exon	Gene	Variant	Variant type	Quality scores	DP	DV	AF in 1000G	AF in ExAC	CADD	CADD_ Phred	PROVEAN score	PolyPhen‐2	MutationTaster
X	12	*FRMD7* (NM_194277.2)	c.1419_1422dup, p.Tyr475fs	Frameshift mutation	222	75	42	NA	NA	1.737553	17.02	−185.593	NA	1, D

Abbreviations: DP, depth of coverage; DV, depth of variant; MutationTasterm, prediction disease causing; NA, not available; PROVEAN, prediction deleterious (cutoff = −2.5).

### 3.2 Clinical manifestation

In our study, we identified a novel *FRMD7* variant (NM_194277.2:c.1419_1422dup, p.Tyr475fs) which may lead to misexpress of FRMD7 protein in a Chinese Han IIN family with manner of X‐linked recessive. The proband was diagnosed with congenital nystagmus without other visual impairment. The onset of nystagmus manifested as an involuntary lateral movement of both eyes, appeared few months after birth, and aggravated when nervous or staring appeared few months. He was disturbed with decreased visual acuity and moderate compensatory but without other features such as squint, hypermetropia, astigmatism, and other symptoms. His daughter (Ⅵ1) carrying this variant has not been affected with ocular problems. Proband's aunt (Ⅳ8) suffered with IIN had very similar manifestations to him.

## 4 DISCUSSION

IIN is generally considered as a common ophthalmic disorder characterized by eye oscillation. The pathogenicity of IIN can be divided into two categories according to the locations of anomalies: (a) sensory deficiency—abnormal information conduction between fovea centralis and cerebrum (afferent nerves) and (b) motor deficiency—abnormal information conduction between ocular movement control center and extrinsic ocular muscles (efferent nerves).^12^ Mutations in *FRMD7*, *GPR143,* and *NYS5* are associated with IIN.

Although the mutations in *FRMD7* are responsible for about 47% of X‐linked nystagmus, pathogenesis of *FRMD7* and the relationship between genotypes and phenotypes remain unclear.^5^ Since the identification of *FRMD7* as a causative gene of IIN, to date, 7 different truncating mutations have been identified in exon 12 of *FRMD7*.^13^, ^14^ In our study, a novel frameshift variant c.1419_1422dup/p.Tyr475fs which led to the premature termination of protein translation at codon 475 was found in exon 12 of *FRMD7* in a Chinese Han family. In addition, this variant, located the high conserved N‐terminal region in FRMD7, plays an important role in nuclear protein localization and during neuronal development through remodeling of the actin cytoskeleton.^15^, ^16^ FRMD7 protein mainly expressed in human mature kidney, pancreas, liver, and low levels in brain and heart. Moreover, FRMD7 protein in brain was limited to regions known as motor control of eye movement as mid‐ and hindbrain.^3^

There are two important structural domains in amino terminal of FRMD7 protein: B41 and FERM‐C, which share close homology with FARP1. As guanine nucleotide exchange factor (GEF) for RAC1, FARP1 plays a key role in semaphorin signaling, dendrite development, and the formation of synapses in related signaling pathways. Similar to FARP1, FRMD7 involves in neurite development through the activation of the GTPase RAC1 and plays a key role in the control of eye movement and gaze stability. Although the variant p.Tyr457fs identified in our study is far from the two important domains known, the truncated protein may destroy the function of FRMD7, which led to the manifestations of nystagmus in the family. In addition, FRMD7 is located in the actin‐rich regions of the neuronal cytoskeleton, in primary neurite as well as actin‐rich cones end of growth.

Most *FRMD7*‐IIN cases, characterized with involuntary eye oscillation caused by selective loss of horizontal optokinetic reflex,^9^ often have visual field defect, so the compensatory head posture is the common accompanied symptom(15% of affected individuals).^17^ In our study, the proband was plagued by nystagmus, which manifested as decreased visual acuity and moderate compensatory but without other features such as squint, hypermetropia, astigmatism, and other symptoms. The penetrance of X‐linked IIN in females was approximately 50% and full in males.^17^ Guo et al (2014) described a Chinese Han family including 4 male patients with IIN and one asymptomatic female carrier.^18^ Tomas et al showed that clinically unaffected females may suffer subclinical form of nystagmus and had slightly better visual acuity than male patients.^19^ But they did not observe significant differences in amplitude, frequency, and waveform of nystagmus between females and males.^17^ The penetrance mechanism can be variant: X‐inactivation, other gene interaction, and environmental factors, but X‐inactivation, which means epigenetic silencing of one X chromosome in each female cell,^20^ is a focused reason.^21^ In our subjects, daughter of proband is a carrier of variant but without eye symptoms, which is the evidence for X chromosome random inactivation.

Considering that IIN affects the quality of life and mental health of patients and the poor intervention effect, molecular analysis and further prenatal genetic screening are of critical significance. It is difficult to determine which gene is associated IIN in the affected individuals based only on clinical manifestations, and the traditional molecular techniques as a Sanger sequencing were usually costly and inefficiently. However, technology development and NGS technique is becoming a helpful tool to identify genes involved in pathogenesis and provide approach to find rare variants in monogenic diseases. NGS also had a higher positive rate and higher efficiency than Sanger sequencing and is widely applicated in the molecular analysis of Mendelian disorders.^22^, ^23^ Our research confirmed that NGS is an exact and efficient diagnostic tool, which enables us identify a novel frameshift *FRMD7* variant in a Chinese IIN family. However, further functional investigation and mutant analysis are needed to reveal the pathogenic mechanisms of IIN caused by p.Tyr475fs variant.
