# Supplementary material for: Next‐generation sequencing identifies a novel frameshift variant in FRMD7 in a Chinese family with idiopathic infantile nystagmus
Source: J Clin Lab Anal. 2019 Sep 8;34(1):e23012. doi: 10.1002/jcla.23012 (PMC6977136; doi:10.1002/jcla.23012)
Supplement: Supplementary file 1 [file JCLA-34-e23012-s001.docx]

**Appendix S1. 239 genes contained in the IIN capture panel**

*ABCA4; ABCB6; ADAM9; AHI1; AIPL1; ARL13B; ARL6; BBS1; BBS10; BBS12; BBS2; BBS4; BBS5; BBS7; BBS9; BCOR; BEST1; C1QTNF5; C2orf71; C8orf37; CA4; CACNA1F; CACNA2D4; CC2D2A; CDH23; CDHR1; CEP290; CEP41; CERKL; CHM; CHMP4B; CLRN1; CNGA1; CNGA3; CNGB1; CNGB3; CNNM4; COL11A1; COL11A2; COL2A1; COL4A1; COL8A2; COL9A1; COL9A2; CRB1; CRX; CRYAA; CRYAB; CRYBA1; CRYBA4; CRYBB1; CRYBB2; CRYBB3; CRYGC; CRYGD; CRYGS; CTDP1; CYP4V2; DFNB31; DHDDS;* *DLAT; EFEMP1; EPHA2; EYS; FAM161A; FBN1; FOXC1; FOXE3; FRMD7; FSCN2; FZD4; GDF6; GJA1; GJA3; GJA8; GNAT1; GNAT2; GPR179; GRK1; GRM6; GUCA1A; GUCA1B; GUCY2D; HCCS; HMX1; HSF4; IDH3B; IMPDH1; IMPG2; IQCB1; KCNJ13; KCNV2; KIF21A; KLHL7; LAMA1; LCA5; LIM2; LRAT; LRP5; LTBP2; MAF; MAK; MERTK; MFRP; MIP; MKKS; MKS1; MYO7A; NDP; NHS; NMNAT1; NPHP1; NPHP4; NR2E3; NRL; NTF4; NYS2, NYS3, NYS4, NYS5, NYS7, NYX; OPA1; OPA3; OPN1LW; OPN1MW; OTX2; PAX6; PCDH15; PDE6A; PDE6B; PDE6C; PDE6G; PDE6H; PDZD7; PITPNM3; PITX2; PITX3; PRCD; PROM1; PRPF3; PRPF31; PRPF6; PRPF8; PRPH2; PRSS56; RAX; RAX2; RB1; RD3; RDH12; RDH5; RGR; RHO; RIMS1; ROM1; RP1; RP2; RP9; RPE65; RPGR; RS1; SAG; SDCCAG8; SEMA4A; SHH; SIX6; SLC24A1; SNRNP200; SOX2; SPATA7; TEAD1; TIMP3; TMEM216; TMEM67; TOPORS; TRIM32; TSPAN12; TTC8; TULP1; USH1C; USH1G; USH2A; VCAN; VSX1; VSX2; ZNF513; ACVRL1; AP3B1; BLOC1S3; BLOC1S6; BMP4; C10orf2; CABP4; CHST6; DCN; DTNBP1; ELOVL4; ENG; FLNA; GPR143; GPR98; GSN; HESX1; HPS1; HPS3; HPS4; HPS5; HPS6; IKBKG; INPP5E; KRT12; KRT3; MC1R; OAT; OCA2; PHOX2A; PIKFYVE; POLG; RAB28; RAB3GAP1; RAB3GAP2; RLBP1; SLC25A4; SLC45A2; SLC4A11; SMAD4; STRA6; TMEM114; TMEM126A; TRPM1; TUBB3; TUBGCP6; TYR; TYRP1; UBIAD1; UNC119; VHL; ZEB1*
